# Peripheral neurotransmitter metabolic signatures in schizophrenia: a targeted metabolomics study

**DOI:** 10.3389/fpsyt.2026.1935417

**Published:** 2026-09-16

**Authors:** Bing Han, Yu Guo, Baie Feng, Mingzhu Zheng, Xianzi Chu, Li Liu, Peng Sun, Xiaoyan Yan, Feng Wang, Tianqi Shen

**Affiliations:** 1 Department of Clinical Laboratory, No. 984 Hospital of the PLA, Beijing, China; 2 Health Service Department, No. 984 Hospital of the PLA, Beijing, China; 3 Department of Respiratory Medicine, No. 984 Hospital of the PLA, Beijing, China; 4 Department of Radiology, No. 984 Hospital of the PLA, Beijing, China; 5 Department of Psychiatry, No. 984 Hospital of the PLA, Beijing, China; 6 Psychiatry and Psychology Center, No. 984 Hospital of the PLA, Beijing, China; 7 Department of Anesthesiology, No. 984 Hospital of the PLA, Beijing, China; 8 Combat Trauma Center, No. 984 hospital of the PLA, Beijing, China

**Keywords:** biomarkers, metabolomics, neurotransmitter agents, schizophrenia, targeted metabolomics

## Abstract

**Objective:**

To investigate peripheral metabolic alterations in patients with schizophrenia using a two-stage metabolomic workflow of untargeted analysis and targeted re-analysis,explore the associated metabolic pathways, identify potential peripheral metabolic biomarkers, and elucidate the underlying metabolic dysregulation mechanisms.

**Methods:**

Plasma samples were collected from 24 schizophrenia patients and 10 healthy controls for non-derivatized targeted metabolomics analysis. Differential metabolites were identified and visualized using box plots. Hierarchical clustering analysis was performed to evaluate expression pattern differences across samples, and KEGG pathway enrichment analysis was conducted to map the involved biological pathways. False discovery rate (FDR) correction was applied for multiple comparisons.

**Results:**

Among the 21 quantified metabolites, four exhibited significant differences after FDR correction (FDR < 0.05). α-Ketoglutarate was significantly elevated in the patient group, whereas succinic acid, tryptamine, and amino butyric acid were significantly reduced. The opposite variations of α-ketoglutarate and succinic acid suggest a peripheral TCA cycle metabolic bottleneck. Hierarchical clustering based on these four metabolites effectively distinguished patients from healthy controls. KEGG enrichment demonstrated that these metabolites converged predominantly on amino acid metabolism, energy metabolism, and most notably the GABAergic synapse pathway, offering peripheral evidence supporting the core position of GABAergic dysfunction in schizophrenia.

**Conclusion:**

Schizophrenia patients display significant peripheral metabolic disturbances characterized by dysregulated amino acid metabolism, synaptic transmission abnormalities, and energy metabolism imbalance. The four-metabolite panel (α-ketoglutarate, succinic acid, tryptamine, and amino butyric acid) may provide experimental basis for further mechanistic research and therapeutic development.

## Introduction

Schizophrenia is a prevalent and severe psychiatric disorder characterized by profound disturbances in perception, cognition, emotion, and behavior (1). Despite decades of research, its precise etiology remains incompletely understood (2). Nevertheless, accumulating evidence has established that dysregulated amino acid metabolism, disrupted central and peripheral neurotransmitter homeostasis, and aberrant mitochondrial energy metabolism are critically involved in the pathophysiological cascade of schizophrenia (3).

Neurotransmitters and their precursor metabolites serve as essential molecular bridges connecting systemic metabolic processes with central nervous system (CNS) function (4). Specifically, classical neurotransmitters—including γ-aminobutyric acid (GABA), glutamate, and serotonin—directly orchestrate synaptic transmission and maintain the excitatory–inhibitory balance in neural circuits (5). Meanwhile, α-ketoglutarate and succinate, as key intermediates of the tricarboxylic acid (TCA) cycle, are indispensable for sustaining cerebral energy supply and mitochondrial bioenergetics (3). Tryptamine, a direct biosynthetic precursor of serotonin, is actively involved in the regulation of tryptophan metabolic pathways (6).

In recent years, metabolomic studies have extensively investigated metabolic alterations in schizophrenia. Prior work has established that (1): GABA levels and GABAergic interneuron function are consistently reduced in schizophrenia brain tissues (7) (2); glutamatergic system dysfunction, particularly NMDA receptor hypofunction, is a well-recognized mechanism (8); and (3) tryptophan metabolism shows a directional shift toward the kynurenine pathway (9). However, most of these studies have relied on untargeted approaches without targeted quantitative validation, and have largely focused on isolated changes in single metabolites or single pathways. In particular, the coordinated alterations of multiple metabolites within the same pathway—and more importantly, opposite-directional changes of different intermediates that may indicate metabolic bottlenecks—have received limited attention. Based on these considerations, we hypothesized that peripheral neurotransmitter and related metabolite alterations in schizophrenia represent a coordinated dysregulation across three functional modules—amino acid metabolism, synaptic signaling, and energy metabolism—with the TCA cycle potentially exhibiting a metabolic bottleneck (opposite changes of α-ketoglutarate and succinate) and the GABAergic synapse pathway serving as a convergence point for multiple metabolite disruptions. The present study aimed to employ targeted metabolomics to re-analysis candidate metabolites identified from prior untargeted screening, and to test whether this coordinated dysregulation pattern is detectable in peripheral blood.

To date, most related investigations have focused on neurotransmitter profiles within brain tissues or cerebrospinal fluid; however, the acquisition of such samples remains notably challenging and highly invasive, thereby limiting their translational applicability (10). In contrast, peripheral venous blood offers substantial practical advantages, including convenient accessibility, minimal patient burden, and compatibility with high-throughput batch analyses. These features render it a promising and pragmatic matrix for the discovery of potential peripheral metabolic biomarkers in schizophrenia (11).

In the present study, we employed a targeted metabolomics approach based on non-derivatized ultra-performance liquid chromatography-tandem mass spectrometry (UPLC-MS/MS) to quantitatively compare peripheral neurotransmitter profiles between schizophrenia patients and healthy controls. Specifically, we aimed to (1): identify differentially expressed neurotransmitters and related metabolites in the peripheral blood of schizophrenia patients following false discovery rate (FDR) correction for multiple comparisons (2); evaluate whether the identified metabolites, as a combined panel, could effectively distinguish patients from healthy controls; and (3) map the involved biological pathways through Kyoto Encyclopedia of Genes and Genomes (KEGG) enrichment analysis. By integrating differential metabolite identification, hierarchical clustering, and KEGG pathway enrichment analysis, we aimed to delineate the characteristic metabolic disturbances associated with schizophrenia and to evaluate the potential utility of peripheral neurotransmitter signatures as auxiliary diagnostic biomarkers, thereby providing experimental evidence for further mechanistic exploration and targeted therapeutic development.

## Methods

### Study design and participants

This targeted metabolomics study was conducted as a targeted re-analysis of untargeted discovery data of our previous metabolomics investigation (12). In the initial screening phase, 30 patients with schizophrenia and 30 healthy controls matched for age, sex, and body mass index (BMI; difference < 1 kg/m²) were enrolled at the No. 984 Hospital of the PLA. Orthogonal partial least-squares discriminant analysis (OPLS-DA) revealed a clear metabolic profile separation between the two groups. For the subsequent targeted quantitative analysis, sample selection was performed by an independent researcher who was not involved in the first-phase non-targeted study. However, because selection was based on OPLS-DA score distribution, which inherently contains group separation information, the selector could not be technically blinded to group assignment. Patient samples were preferentially selected from those with scores concentrated in the central region of the OPLS-DA distribution to minimize the influence of outliers, while control samples were randomly selected from the healthy individuals. Due to limited research funding, only a subset of samples could be analyzed, ultimately yielding 24 patient samples and 10 healthy control samples for targeted quantitative analysis.

All 24 patients in the schizophrenia group met the diagnostic criteria for schizophrenia according to the International Classification of Diseases, 10th Revision (ICD-10), and had been screened to exclude (1): neurological disorders or other psychiatric conditions (2); history of major physical illnesses (3); substance (drugs, alcohol, etc.) abuse or dependence (4); concurrent endocrine disorders including diabetes, hypertension, or hyperlipidemia; and (5) pregnancy or lactation. The patient group comprised 14 males and 10 females, with a mean age of 46.25 ± 9.44 years,with a mean Brief Psychiatric Rating Scale (BPRS) score of 39.50 ± 6.43 and a mean Positive and Negative Syndrome Scale (PANSS) score of 96.0 (86.75, 102.75). The healthy control group comprised 7 males and 3 females, with a mean age of 43.36 ± 8.76 years. Symptom severity was assessed independently by two senior psychiatrists using the BPRS and PANSS.

All participants provided written informed consent, and the study protocol was approved by the Ethics Committee of the No. 984 Hospital of PLA (approval number: 20260311-01; date: March 11^th^ 2026).

### Preparation of standard solutions

Mixed stock standard solutions were prepared by accurately weighing reference standards and dissolving them in appropriate solvents. A series of calibration working solutions were obtained by stepwise gradient dilution. Each calibration standard was processed following the same procedure as the samples prior to UPLC-MS/MS analysis, and the standard calibration curves were constructed based on the peak areas versus nominal concentrations.

### UPLC-MS/MS analysis

#### Chromatographic conditions

Chromatographic separation was performed on an Agilent 1290 Infinity LC system. The samples were maintained at 4°C in the autosampler, and the column oven temperature was set at 35°C. Separation was achieved on an ACQUITY UPLC BEH Amide column (1.7 μm, 2.1 mm × 150 mm; Waters). The mobile phase consisted of (A) 25 mM ammonium formate in water containing 0.1% formic acid and (B) acetonitrile. The flow rate was 300 μL/min, and the injection volume was 1 μL. The gradient elution program was as follows: 0-1 min, 90% B; 1-16 min, linear decrease from 90% to 50% B; 16–18 min, 50% B; 18–18.1 min, linear increase from 50% to 90% B; and 18.1-23 min, 90% B. Quality control (QC) samples, prepared by pooling equal volumes of all individual samples, were injected at regular intervals throughout the analytical sequence to monitor system stability and method reproducibility. A mixture of reference standards was also interspersed to calibrate chromatographic retention times. The detailed instrument specifications are listed in **Supplementary Table 1**.

#### Mass spectrometric conditions

Mass spectrometric detection was carried out on a SCIEX 6500 + QTRAP triple quadrupole mass spectrometer equipped with an electrospray ionization (ESI) source operating in negative ion mode. Data acquisition was performed in multiple reaction monitoring (MRM) mode. The source parameters were optimized as follows: source temperature, 450°C; ion source gas 1 (Gas1), 45; ion source gas 2 (Gas2), 45; curtain gas (CUR), 30; and ion spray voltage floating (ISVF), −4500 V. Each metabolite was identified and quantified based on its specific precursor- to -product ion transitions and retention time matching with reference standards.

### Method validation

Limits of detection and quantification: The limit of detection (LOD) was defined as the concentration corresponding to a signal-to-noise ratio (S/N) of 3, representing the lowest analyte concentration distinguishable from baseline noise in the sample matrix. The limit of quantification (LOQ) was defined as the concentration corresponding to an S/N of 10, representing the lowest concentration that could be accurately quantified by the method.

Precision: Method precision was evaluated by calculating the relative standard deviations (RSDs) of the QC samples injected repeatedly throughout the analytical run.

### Statistical analysis

Statistical analyses were performed using R software (version 4.4.2). Normality of continuous variables was assessed using the Shapiro-Wilk test. Normally distributed continuous variables were expressed as mean ± standard deviation (SD), non-normally distributed variables as median with interquartile range (IQR), and categorical variables as frequencies (percentages). Chromatographic peak area and retention time data were extracted using Multiquant 3.0.2 software. Metabolite identification was achieved by matching retention times with reference standards, and absolute concentrations were calculated based on standard calibration curves. Group comparisons were conducted using Student’s t-test.

To evaluate the discriminatory performance of the identified metabolites, univariate and multivariable receiver operating characteristic (ROC) analyses were performed. Area under the curve (AUC) with 95% confidence intervals (CI) were calculated. For multivariable models adjusting for age and sex, Firth penalized logistic regression was applied due to the small sample size and the presence of complete separation in some models, which precluded standard maximum likelihood estimation. Model performance was further evaluated using leave-one-out cross-validation (LOOCV), in which each sample was iteratively left out as the test set while the remaining samples were used for model training. The LOOCV-estimated AUC was calculated to assess the generalizability of the model.

To explore the relationship between neurotransmitter concentrations and clinical symptom severity, Pearson correlation analyses were performed between the levels of differential metabolites and BPRS scores within the patient group. All statistical tests were two-sided, and a P-value < 0.05 was considered statistically significant. For multiple comparisons, false discovery rate (FDR) correction was applied using the Benjamini-Hochberg method where appropriate.

## Results

### Method validation and quality control

The representative XIC chromatogram of mixed standards showed satisfactory chromatographic separation of the target metabolites, with symmetrical peak shapes and adequate resolution for quantitative analysis. Method validation demonstrated that all calibration curves exhibited excellent linearity over the concentration ranges covering the sample measurements, with correlation coefficients (R) exceeding 0.99 for all target metabolites. The relative standard deviations (RSDs) of QC samples were all below 30% (**Figure 1**), confirming acceptable instrumental stability and method reproducibility. Collectively, these quality control metrics met the requirements for targeted metabolomics analysis and ensured the reliability of subsequent quantitative comparisons.

**Figure 1:**
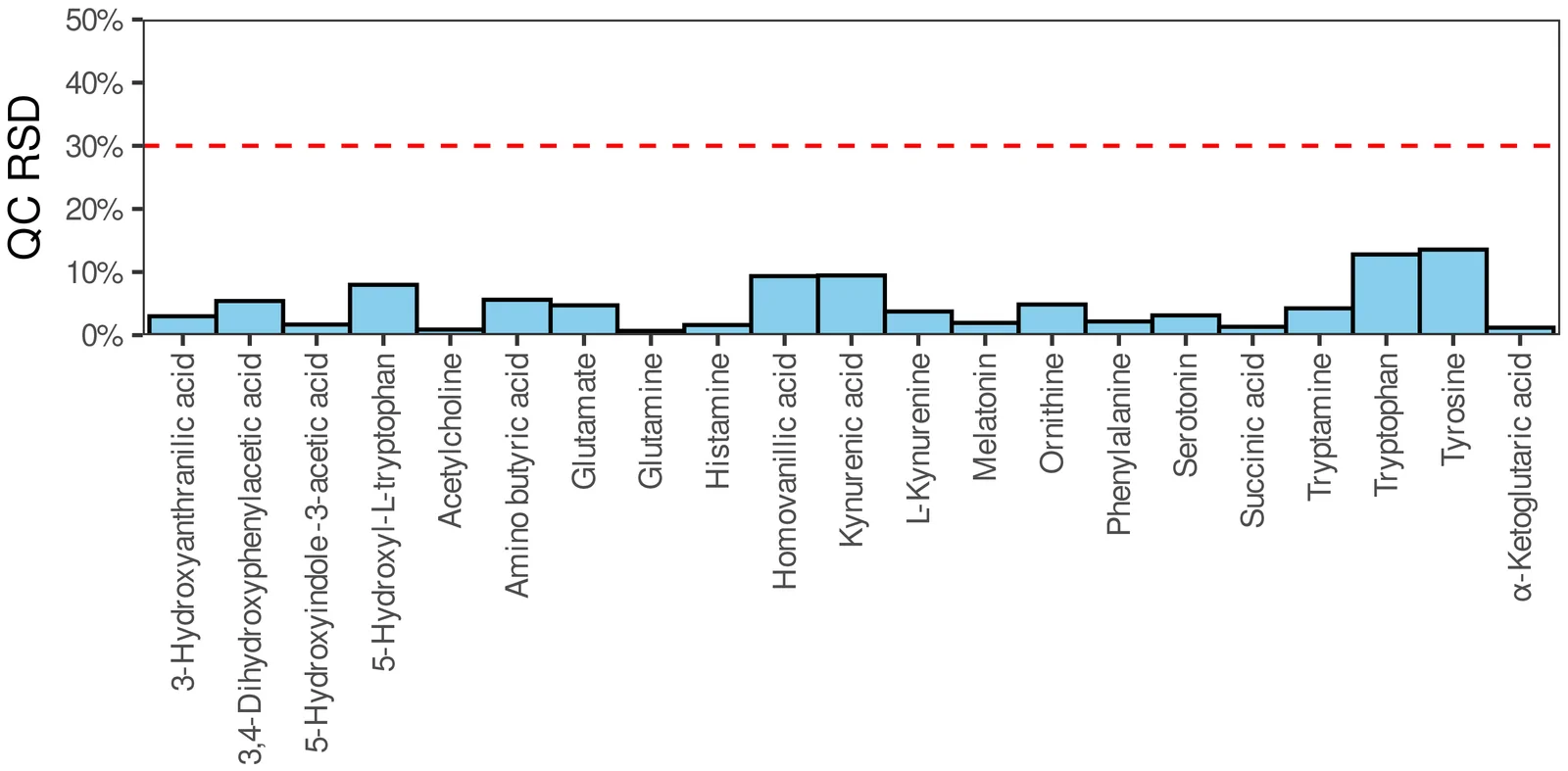
RSDs of QC samples for the target metabolites.

### Identification of differential neurotransmitters

Targeted metabolomics analysis quantified 33 neurotransmitters and related derivatives, of which 21 were reliably detected in plasma samples. Initial screening using Student’s t-test revealed six metabolites with significantly different levels between the patient and control groups (raw P < 0.05): α-Ketoglutaric acid, succinic acid, tryptamine, amino butyric acid, serotonin and glutamate. Among these, tryptamine, succinic acid, and amino butyric acid showed decreasing trends in the patient group.

To control for false discovery rates due to multiple comparisons, FDR correction was applied using the Benjamini–Hochberg method. After correction, four metabolites remained statistically significant (FDR < 0.05): α-ketoglutarate was significantly elevated in the patient group (FoldChange = 4.022, FDR < 0.001), whereas succinic acid (FoldChange = 0.268, FDR < 0.001), tryptamine (FoldChange = 0.913, FDR = 0.002), and amino butyric acid (FoldChange = 0.924, FDR = 0.016) all exhibited decreasing trends. Glutamate (FDR = 0.054) and serotonin (FDR = 0.148), despite having raw P values below 0.05, did not survive FDR correction (FDR > 0.05), suggesting that their observed differences may be influenced by multiple comparison effects and warrant further validation in larger cohorts.

As shown in **Figure 2A**, the four FDR-significant metabolites displayed clear distribution differences between the two groups; **Figure 2B** presents the overall ranking of the 21 quantified metabolites, with the four FDR < 0.05 metabolites positioned above the significance threshold. All quantified metabolite data are summarized in **Supplementary Table 2**.

**Figure 2:**
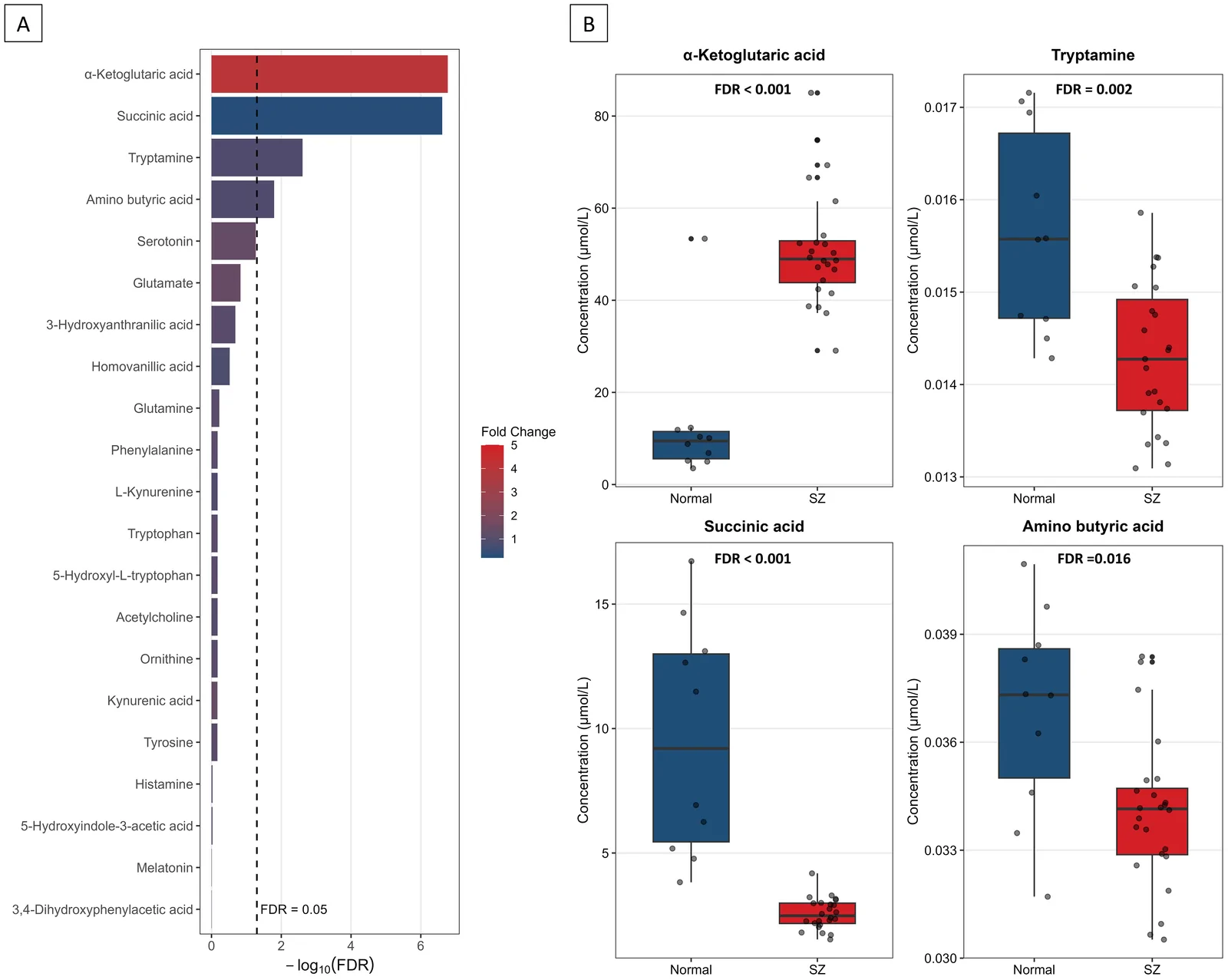
Differential metabolites identified by targeted metabolomics. **(A)** Statistical bar chart of differential metabolites between SZ and Normal groups; **(B)** Box‑plots showing concentrations of key differential metabolites in SZ patients and normal controls.

To further evaluate the discriminatory ability of the four metabolites and their combination, univariate and multivariable ROC curves were generated. Univariate ROC analysis yielded AUC values of 0.925 (95% CI: 0.777–1.000), 0.984 (95% CI: 0.996–1.000), 0.838 (95% CI: 0.691–0.984), and 0.775 (95% CI: 0.563–0.995) for α-ketoglutaric acid, succinic acid, tryptamine, and amino butyric acid, respectively. After adjustment for age and sex in multivariable logistic regression models, the AUCs for α-ketoglutaric acid and succinic acid increased to 0.967 (95% CI: 0.899–1.000) and 1.000 (95% CI: 1.000–1.000). Tryptamine and amino butyric acid also exhibited increasing AUC trends, but the models did not converge due to complete separation, rendering the results unreliable. LOOCV further supported the stability of the four-metabolite panel, yielding AUCs of 0.908, 1.000, 0.775, and 0.717 for the four metabolites. These results indicate that the four-metabolite panel shows good potential for discriminating patients from healthy controls (**Supplementary Figure 1**).

### Hierarchical clustering analysis

As shown in **Figure 3**, the sample dendrogram at the top clearly partitioned all samples into two major clusters, which largely corresponded to the Normal and SZ groups, indicating that the combined expression profile of the four FDR-significant metabolites effectively discriminated between the two groups.

**Figure 3:**
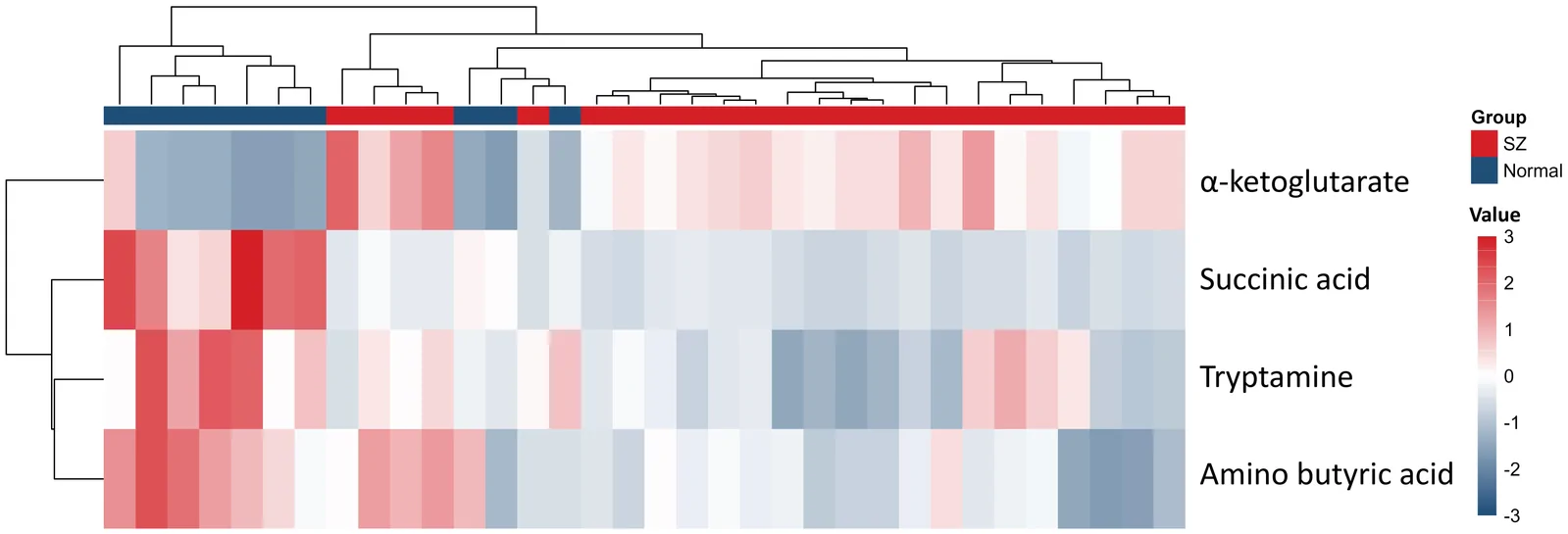
Heatmap of differential metabolite expression patterns.

In terms of color distribution, the patient cluster was characterized by red (high expression) for α-ketoglutarate, whereas succinic acid, tryptamine, and amino butyric acid exhibited blue-purple (low expression) in the SZ group; the Normal cluster displayed the opposite pattern. This clustering pattern was fully consistent with the FDR-corrected group comparison results in the preceding section, further validating the altered expression of these four metabolites in the peripheral blood of schizophrenia patients.

Of note, a few cross-cluster observations were noted: several patient samples fell within the control cluster, and a few control samples clustered with the patient group, suggesting that inter-individual biological variation exists. Nevertheless, the combined expression profile of the four metabolites still effectively distinguished the two groups, supporting their potential utility as a biomarker panel.

### KEGG pathway enrichment analysis

To explore the biological processes involving the four FDR-significant differential metabolites (α-ketoglutarate, succinic acid, tryptamine, and amino butyric acid), KEGG pathway enrichment analysis was performed. The results showed that the four differential metabolites were mapped to 63 KEGG canonical pathways, of which 45 pathways reached statistical significance (FDR < 0.05).

As shown in **Figure 4**, among the top 20 pathways ranked by significance, the GABAergic synapse pathway exhibited the smallest FDR value and the highest Rich Factor, indicating that this pathway is the most profoundly affected metabolic pathway in schizophrenia. Furthermore, the Alanine, aspartate and glutamate metabolism, Butanoate metabolism, and Synaptic vesicle cycle pathways also achieved highly significant enrichment (FDR < 0.05, Rich Factor > 0.2), suggesting that disturbances in amino acid metabolism, energy metabolism, and synaptic dysfunction are important pathological features of schizophrenia. Details of all involved KEGG pathways are presented in **Supplementary Table 3**.

**Figure 4:**
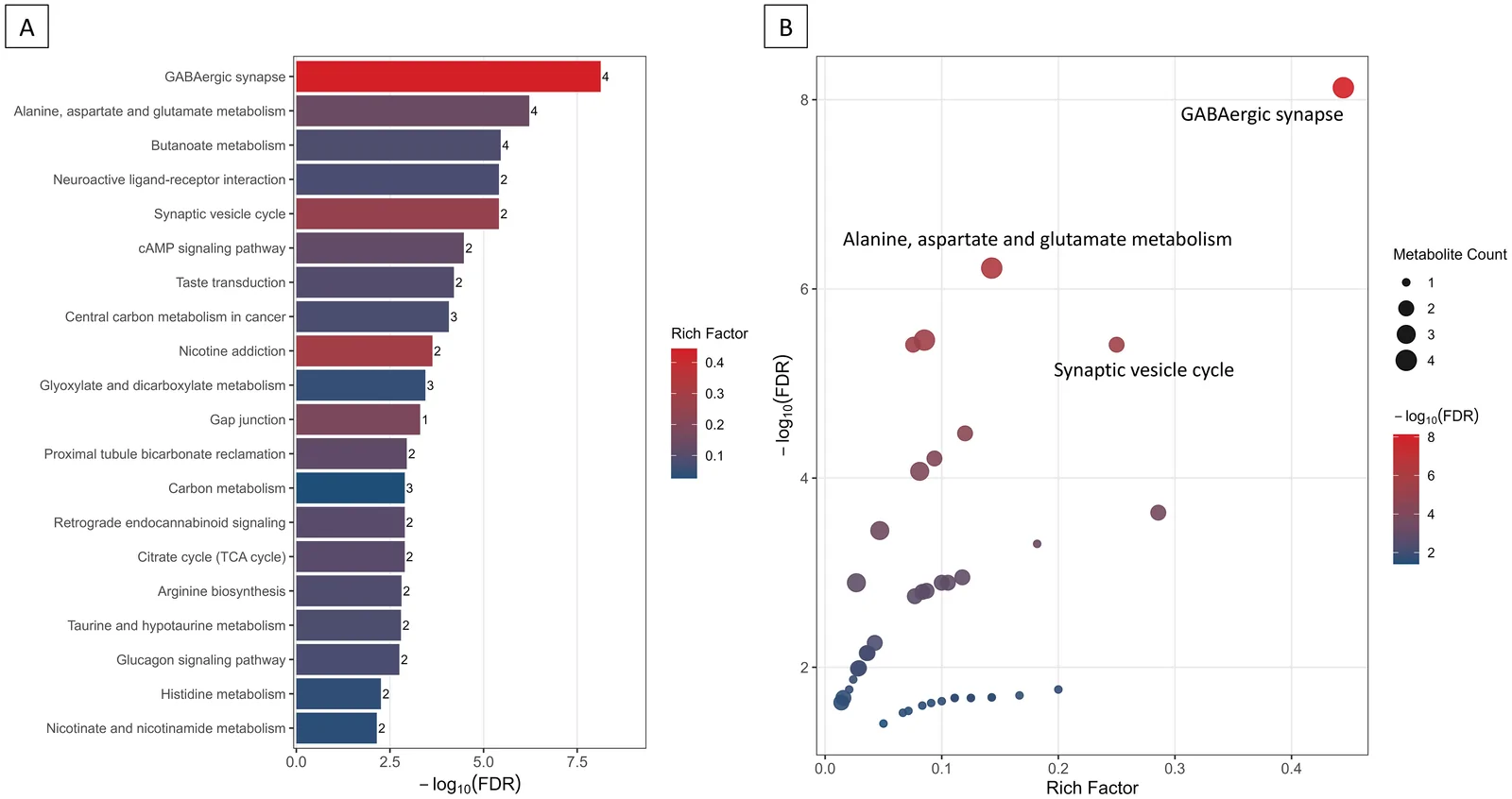
KEGG enrichment analysis of differential metabolites. **(A)** Bar plot of KEGG pathway enrichment for differential metabolites; **(B)** Bubble plot of KEGG pathway enrichment for differential metabolites.

### Correlation between metabolite concentrations and clinical symptoms

To explore the association between the four FDR-significant metabolites and disease severity, Pearson correlation analyses were performed between metabolite concentrations and BPRS scores as well as PANSS total scores within the patient group (n = 24). The results showed that none of the four metabolites exhibited a significant correlation with BPRS scores or PANSS scores (all P > 0.05; **Figure 5**).

**Figure 5:**
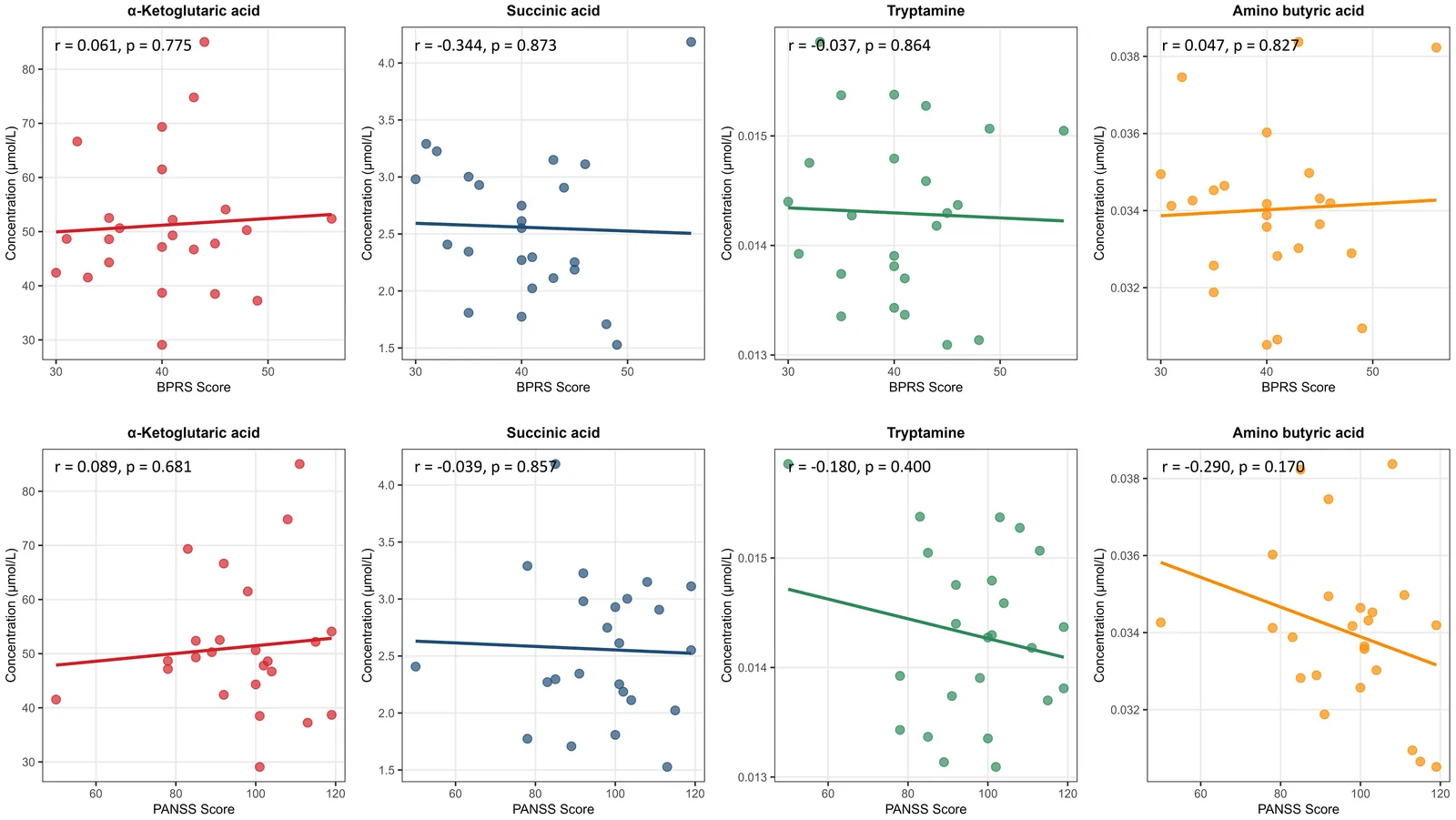
Correlation between metabolite concentrations and BPRS and PANSS in schizophrenia patients.

These negative findings may be attributed to the limited sample size (n = 24), suggesting insufficient statistical power to detect moderate-sized correlations. It also suggests that these peripheral metabolites may primarily reflect disease “state” rather than “severity,” and their relationship with clinical symptoms warrants further investigation in larger cohorts.

## Discussion

The pathophysiology of schizophrenia involves the interplay of multiple biological processes, including neurotransmitter imbalance, mitochondrial energy dysfunction, and amino acid metabolic disturbances. However, the precise manner in which these metabolic networks are reflected in peripheral blood remains incompletely understood (13, 14). Notably, peripheral metabolic profiles are segregated from central nervous system status by the blood–brain barrier, and peripheral metabolite alterations cannot be directly equated to central pathophysiological changes. In the present study, we employed targeted metabolomics to systematically compare the quantitative profiles of neurotransmitters and related metabolites in the peripheral blood of schizophrenia patients and healthy controls. Four metabolites—α-ketoglutarate, succinic acid, tryptamine, and amino butyric acid—exhibited significant differences between the two groups (FDR < 0.05), with α-ketoglutarate being significantly elevated and succinic acid, tryptamine, and amino butyric acid significantly reduced. KEGG pathway enrichment analysis further revealed that these metabolites are predominantly involved in pathways related to amino acid metabolism, synaptic signaling, and energy metabolism. Collectively, these findings identify a systemic peripheral metabolic disturbance centered on the amino acid-neurotransmitter-energy metabolism axis in schizophrenia patients, which may serve as a peripheral metabolic correlate of disease pathophysiology rather than a direct mirror of central dysfunction.

α-Ketoglutarate is a key intermediate of the TCA cycle and a critical node in glutamate metabolism (15). In this study, plasma α-ketoglutarate concentrations were significantly elevated in patients compared with healthy controls (FoldChange = 4.022, FDR < 0.001). This upregulation is consistent with previous metabolomic findings in brain tissues and cerebrospinal fluid from schizophrenia patients (16), implying a potential association between peripheral TCA cycle dysregulation and central metabolic abnormalities. However, considering the restriction of the blood–brain barrier, the peripheral accumulation of α-ketoglutarate cannot directly represent central TCA cycle metabolic bottlenecks or feedback dysregulation (13). Additionally, peripheral α-ketoglutarate elevation may be indirectly associated with central glutamatergic dysfunction, which is consistent with the glutamate hypothesis of schizophrenia only in a correlative manner (14). The significant enrichment of the “alanine, aspartate and glutamate metabolism” pathway (FDR < 0.05) further supports the notion that coupled disturbances in amino acid and energy metabolism represent a key pathophysiological feature of schizophrenia.

Succinic acid, another key intermediate of the TCA cycle (17), exhibited a significant decrease in this study (FoldChange = 0.268, FDR < 0.001). In contrast to the upregulation of α-ketoglutarate, the reduction of succinic acid suggests a coexisting pattern of “congestion and depletion” within the TCA cycle—where some intermediates accumulate due to upstream blockade while downstream metabolites are excessively consumed due to impaired mitochondrial oxidative phosphorylation. This pattern is a hallmark of mitochondrial dysfunction (18). Given the high energy dependence of neurons on mitochondrial oxidative phosphorylation, central mitochondrial damage is closely linked to schizophrenia-related neurodegeneration and cognitive impairment (16). Nevertheless, the observed peripheral TCA cycle disruption is only a systemic metabolic manifestation of schizophrenia, and its direct correspondence to central neuronal energy metabolism dysfunction cannot be confirmed in the present study.

Tryptamine is a key intermediate in the tryptophan metabolic pathway and the direct biosynthetic precursor of serotonin (5-HT) (19). In this study, plasma tryptamine levels were significantly lower in patients than in healthy controls (FoldChange = 0.913, FDR = 0.002), suggesting a directional shift in tryptophan metabolis-with tryptophan being preferentially channeled toward the kynurenine pathway rather than the 5-HT synthesis pathway. Previous studies have reported aberrant activation of the tryptophan-kynurenine pathway in the central nervous system of schizophrenia patients, leading to the accumulation of neurotoxic metabolites such as quinolinic acid, accompanied by reduced 5-HT synthesis (20). The peripheral downregulation of tryptamine may serve as a peripheral reflection of this central metabolic shift, supporting the tryptophan-kynurenine imbalance hypothesis of schizophrenia (21). The significant enrichment of the “butanoate metabolism” pathway further suggests that gut-microbiota-derived tryptophan metabolites may also contribute to the pathology. This possibility is supported by recent multi-kingdom microbiome findings in depression, linking tryptophan-related microbial metabolites to cognitive function and symptom severity, suggesting that gut microbial dysregulation may contribute to psychiatric metabolic disturbances through the microbiota-gut-brain axis (22).

Amino butyric acid, the peripheral metabolite corresponding to the major central inhibitory neurotransmitter GABA, showed a significant decrease in this study (FoldChange = 0.924, FDR = 0.016). GABAergic system dysfunction is a well-established central pathophysiological hypothesis of schizophrenia (20). Consistent with reduced central GABA levels reported previously, peripheral amino butyric acid was decreased in patients, suggesting a correlative association between peripheral amino butyric acid alteration and central GABAergic dysfunction, rather than a one-to-one correspondence. Peripheral GABA reduction may indirectly correlate with cortical excitation-inhibition imbalance, which is closely related to schizophrenia positive symptoms (21). The significant enrichment of the “GABAergic synapse” pathway further confirms the peripheral metabolic perturbation of GABA-related signaling in schizophrenia.

KEGG pathway enrichment analysis revealed that the four differential metabolites mapped to 64 canonical pathways, of which 45 reached statistical significance (FDR < 0.05), confirming that these metabolites are not altered in isolation but are involved in the coordinated dysregulation of multiple cellular metabolic and signaling pathways. Although glutamate did not survive FDR correction (FDR = 0.054), its role as a central hub in amino acid metabolism networks-participating in numerous pathways-warrants attention given its proximity to the significance threshold.

Notably, the present study identified a four-metabolite. Previous peripheral metabolomic investigations of schizophrenia have primarily highlighted lipid metabolism disorder, generalized amino acid dysregulation, and unspecified TCA cycle perturbation, with considerable inconsistency in the expression patterns of neurotransmitter and energy metabolites. For example, prior untargeted studies mostly reported decreased peripheral α-ketoglutarate or failed to detect significant tryptamine alterations in schizophrenia patients (23), which is inconsistent with our FDR-validated results of elevated α-ketoglutarate and reduced tryptamine. Additionally, most earlier biomarker studies focused on non-specific systemic metabolites such as glycerate, arachidonic acid, and arginine, which lack direct relevance to schizophrenia core neuro-pathological mechanisms (23). In contrast, the four metabolites identified in this study form a unique peripheral metabolic panel characterized by increased α-ketoglutarate and decreased succinic acid, tryptamine, and amino butyric acid. This signature specifically converges on the integrated amino acid-neurotransmitter-energy metabolism axis, simultaneously linking mitochondrial TCA cycle dysfunction, tryptophan-kynurenine pathway imbalance, and GABAergic synaptic impairment-three pivotal but previously isolated pathophysiological hypotheses of schizophrenia. Compared with fragmented metabolic markers reported in previous studies, this coordinated metabolite panel exhibits higher neurobiological specificity and better explains the systemic synaptic and energetic dysfunction underlying schizophrenia.

The significantly enriched pathways were predominantly clustered into three functional modules: amino acid metabolism (including alanine, aspartate and glutamate metabolism and butanoate metabolism), synaptic signaling (GABAergic and glutamatergic synapses, and synaptic vesicle cycle), and energy metabolism (TCA cycle). These three modules are not independent; they are tightly interconnected through metabolite–enzyme–signaling networks. Dysregulated amino acid metabolism directly drives imbalances in neurotransmitter (GABA, glutamate, and 5-HT) synthesis (24); impaired synaptic signaling disrupts neurotransmitter release, recognition, and transmission (25); and mitochondrial energy deficits fail to provide sufficient ATP to sustain these highly energy-demanding neural activities. These processes interact reciprocally, forming a vicious cycle of systemic metabolic disturbance in schizophrenia. This coordinated dysregulation pattern aligns with the framework of the BIGHI cohort (26), which has demonstrated systematic interconnections among brain function, peripheral biomarkers, and gut microbiome in psychiatric disorders. Our findings add a peripheral metabolic layer to this framework, supporting the need for multi-omics integration in future investigations.

Of particular interest is the significant enrichment of the “synaptic vesicle cycle” pathway. This cycle is a critical process governing neurotransmitter release, reuptake, and recycling, and its functional integrity directly determines synaptic transmission efficiency and plasticity (27). The enrichment of this pathway suggests impaired vesicular trafficking, fusion, and recycling at presynaptic terminals in schizophrenia patients, consistent with previous reports of abnormal presynaptic protein expression and reduced synapse numbers in this population (28).

Within the patient group (n = 24), we examined the correlations between the four differential metabolite concentrations and BPRS and PANSS total scores. The results showed that none of the metabolites exhibited a significant correlation with either clinical scale (P > 0.05 for all). Potential explanations include (1): the relatively small sample size, which limited statistical power (2); the possibility that these peripheral metabolites serve as classifiers distinguishing diseased from non-diseased states rather than quantitative indicators of symptom severity; and (3) the confounding effect of antipsychotic treatment, which may have obscured genuine metabolite-symptom associations. This interpretation aligns with a recently proposed framework conceptualizing disease as a systematic deviation from a healthy ecological baseline, where certain biomarkers may discriminate health from disease without linearly reflecting symptom severity (29).

Several limitations of this study should be acknowledged. First, the sample size is relatively small—24 patients and 10 controls. Although targeted metabolomics generally requires smaller sample sizes than untargeted approaches, the limited cohort still restricts statistical power, particularly for correlation analyses (where only strong effects could be detected in 24 patients) and precludes stratified subgroup analyses (e.g., by disease duration or medication type). Second, all patients were under antipsychotic treatment at the time of sample collection. Antipsychotic agents are known to affect dopaminergic, serotonergic, and glutamatergic systems, and may also interfere with mitochondrial energy metabolism. Therefore, the observed metabolic differences may partly reflect medication effects rather than being solely attributable to schizophrenia pathology per se. Future studies incorporating drug-naïve or first-episode patients are required to distinguish disease-intrinsic metabolic changes from treatment-related effects. Third, the cross-sectional design cannot establish causality between metabolic abnormalities and schizophrenia pathogenesis, an often overlooked limitation of the present study. All detected peripheral metabolic alterations are observational correlates instead of causal indicators. Single-time-point blood sampling fails to differentiate whether such metabolic dysfunction represents primary pathogenic factors, concurrent disease biomarkers, or secondary adaptive changes induced by chronic illness, neuronal damage or worsening clinical symptoms. This inherent limitation restricts causal interpretation of our findings. Future prospective longitudinal studies with serial metabolite measurements are necessary to clarify the temporal order and causal relationship between peripheral metabolic disturbances and schizophrenia development. Fourth, the link between peripheral metabolites and central nervous system conditions is unclear. Constrained by the blood–brain barrier, blood metabolite concentrations cannot accurately reflect cerebral neurotransmitters and synaptic metabolic function. Inferences connecting peripheral metabolic profiles to central pathological mechanisms herein are merely speculative correlations instead of solid conclusions. Further multi-omics studies combining blood testing, cerebrospinal fluid detection and neuroimaging may verify the peripheral-central metabolic association in schizophrenia.

## Conclusion

In summary, this targeted metabolomics study identified a panel of four peripheral metabolites—α-ketoglutarate, succinic acid, tryptamine, and amino butyric acid—that significantly distinguish schizophrenia patients from healthy controls, with α-ketoglutarate being elevated and the other three reduced in the patient group. These metabolites are predominantly involved in the coordinated dysregulation of amino acid metabolism, GABAergic synaptic signaling, and energy metabolism, highlighting the systemic nature of metabolic disturbances in schizophrenia. Although the limited sample size precludes definitive clinical application at this stage, the four-metabolite panel shows promise as a candidate auxiliary diagnostic biomarker set. Future studies with larger, independent cohorts, particularly incorporating drug-naive patients and longitudinal designs, are warranted to validate these findings and to clarify the causal relationships between peripheral metabolic alterations and the pathophysiology of schizophrenia.

## Data Availability

The datasets presented in this study can be found in online repositories. The names of the repository/repositories and accession number(s) can be found in the article/**Supplementary Material**.

## Supplementary material

Supplementary Figure 1 Univariate and multivariable ROC curves of the four metabolites.

## References

[1] Oatu A Capatina T-F Mandras I-C Comsa A-L Trifu S Pietreanu A-C . Emerging neurobiological and therapeutic insights into schizophrenia: A comprehensive review. Int J Mol Sci. (2026) 27:1906. doi: 10.3390/ijms27041906 41752042 PMC12940323

[2] So M-W Ullah A Waris A Alhumaydhi FA . The etiological role of impaired neurogenesis in schizophrenia: Interactions with inflammatory, microbiome and hormonal signaling. Int J Mol Sci. (2025) 26:9814. doi: 10.3390/ijms26199814 41097077 PMC12524466

[3] Ricci V Martinotti G Mosca A Maina G . Bioenergetic impairment in schizophrenia: Role of mitochondrial signaling in synaptic dysfunction - a systematic review. Front Cell Dev Biol. (2026) 14:1740079. doi: 10.3389/fcell.2026.1740079 41836300 PMC12982393

[4] Sivamaruthi BS Kesika P Chaiyasut C Ragu Varman D . Microbiome driven modulation of neurotransmitters: Implications for neurotransmission and mood disorders. Front Microbiol. (2026) 17:1750377. doi: 10.3389/fmicb.2026.1750377 42131202 PMC13160800

[5] Kandilakis CL Papatheodoropoulos C . Psychedelic modulation of excitation/inhibition balance: A dual-phase neurodevelopmental model. ACS Chem Neurosci. (2026) 17:335–51. doi: 10.1021/acschemneuro.5c00892 41498818 PMC12828726

[6] Shi C Dong J Hui X Xu Z Zhao Z Dong L . Production, mechanisms, and therapeutic strategies of tryptophan metabolites in CNS diseases. Mol Neurobiol. (2025) 63:226. doi: 10.1007/s12035-025-05538-5 41324859 PMC12669377

[7] de Jonge JC Vinkers CH Hulshoff Pol HE Marsman A . GABAergic mechanisms in schizophrenia: Linking postmortem and *in vivo* studies. Front Psychiatry. (2017) 8:118. doi: 10.3389/fpsyt.2017.00118 28848455 PMC5554536

[8] Moghaddam B Javitt D . From revolution to evolution: The glutamate hypothesis of schizophrenia and its implication for treatment. Neuropsychopharmacology. (2012) 37:4–15. doi: 10.1038/npp.2011.181 21956446 PMC3238069

[9] Kynurenine Pathway of Tryptophan Metabolism in Neuropsychiatric Disorders: Pathophysiologic and Therapeutic Considerations - Pubmed. Available online at: https://libvpn2.tmmu.edu.cn:8443/s/gov/nih/nlm/ncbi/pubmed/G.https/33124585/?;x-chain-id=bnbuxwf5jnr4 (Accessed August 1, 2026). 10.9758/cpn.2020.18.4.507 PMC7609208 33124585

[10] Wang J-L Zhao Q Zheng R Fang Y Cao J Zhang B-R . Cerebrospinal fluid and plasma metabolites in Parkinson’s disease: A Mendelian randomization study. Sci Rep. (2026) 16:9588. doi: 10.1038/s41598-025-30521-4 41872227 PMC13009158

[11] Krzyściak W Bystrowska B Karcz P Chrzan R Bryll A Turek A . Association of blood metabolomics biomarkers with brain metabolites and patient-reported outcomes as a new approach in individualized diagnosis of schizophrenia. Int J Mol Sci. (2024) 25:2294. doi: 10.3390/ijms25042294 38396971 PMC10888632

[12] Han B Feng B Ge Y Wang T Liu L Sun P . Untargeted metabolomics reveals plasma metabolic dysregulation in schizophrenia. BMC Psychiatry. (2026). doi: 10.1186/s12888-026-08441-y 42768336 PMC13591716

[13] Flannery RB Flannery GJ . Characteristics of international assaultive psychiatric patients: Review of published findings, 2017-2022. Psychiatr Q. (2023) 94:559–68. doi: 10.1007/s11126-023-10050-x 37667138

[14] Khatami SH Anoosheh S Khodaparast M Maghsoudloonejad A Dadgostar E Asadi A . Multi-omics biomarkers in psychiatric disorders diagnosis and stratification. Clin Chim Acta. (2026) 585:120887. doi: 10.1016/j.cca.2026.120887 41655615

[15] Andersen JV . The glutamate/GABA-glutamine cycle: Insights, updates, and advances. J Neurochem. (2025) 169:e70029. doi: 10.1111/jnc.70029 40066661 PMC11894596

[16] Liu L Zhang W Liu T Tan Y Chen C Zhao J . The physiological metabolite α-ketoglutarate ameliorates osteoarthritis by regulating mitophagy and oxidative stress. Redox Biol. (2023) 62:102663. doi: 10.1016/j.redox.2023.102663 36924682 PMC10026041

[17] Bubber P Hartounian V Gibson GE Blass JP . Abnormalities in the tricarboxylic acid (TCA) cycle in the brains of schizophrenia patients. Eur Neuropsychopharmacol. (2011) 21:254–60. doi: 10.1016/j.euroneuro.2010.10.007 21123035 PMC3033969

[18] Cai J Xiao J Long Y Cao T Lin C Hei G . Metformin-improved cognitive impairment in patients with schizophrenia is correlated with activation of tricarboxylic acid cycle and restored functional connectivity of hippocampus. BMC Med. (2025) 23:349. doi: 10.1186/s12916-025-04218-4 40597190 PMC12211882

[19] Holeček M . Serotonin, kynurenine, and indole pathways of tryptophan metabolism in humans in health and disease. Nutrients. (2026) 18:507. doi: 10.3390/nu18030507 41683329 PMC12899273

[20] Meier TB Savitz J . The kynurenine pathway in traumatic brain injury: Implications for psychiatric outcomes. Biol Psychiatry. (2022) 91:449–58. doi: 10.1016/j.biopsych.2021.05.021 34266671 PMC8630076

[21] Erhardt S Schwieler L Imbeault S Engberg G . The kynurenine pathway in schizophrenia and bipolar disorder. Neuropharmacology. (2017) 112:297–306. doi: 10.1016/j.neuropharm.2016.05.020 27245499

[22] Chen S Zhu B Lu X Huang Y Wang S Wang W . Integrative multi-kingdom gut microbiome analysis uncovers clinical signatures of major depressive disorder. J Affect Disord. (2026) 408:121858. doi: 10.1016/j.jad.2026.121858 42035799

[23] Yao JK Dougherty GG Reddy RD Keshavan MS Montrose DM Matson WR . Altered interactions of tryptophan metabolites in first-episode neuroleptic-naive patients with schizophrenia. Mol Psychiatry. (2010) 15:938–53. doi: 10.1038/mp.2009.33 19401681 PMC2953575

[24] Fujihara K . Beyond the γ-aminobutyric acid hypothesis of schizophrenia. Front Cell Neurosci. (2023) 17:1161608. doi: 10.3389/fncel.2023.1161608 37168420 PMC10165250

[25] Gupta R Jha NK Kumar N Nagraik R Ravi K . From synapse to system: Mechanistic pathways of neural signaling dysfunction in psychiatric disorders. Front Cell Dev Biol. (2026) 14:1762930. doi: 10.3389/fcell.2026.1762930 41799440 PMC12963065

[26] Wu F Zhu B Feng S Li H Zhou J Ning Y . The Brain-Gut Health Initiative (BIGHI): A prospective cohort on psychiatric disorders in China. Res (Wash D C). (2026) 9:1142. doi: 10.34133/research.1142 41783048 PMC12953926

[27] Mochida S . Mechanisms of synaptic vesicle exo- and endocytosis. Biomedicines. (2022) 10:1593. doi: 10.3390/biomedicines10071593 35884898 PMC9313035

[28] Egbujo CN Sinclair D Hahn C-G . Dysregulations of synaptic vesicle trafficking in schizophrenia. Curr Psychiatry Rep. (2016) 18:77. doi: 10.1007/s11920-016-0710-5 27371030 PMC5134737

[29] Zhu B Chen S Diao Y Wang W Huang Y Liang L . Dissecting the ecological structure of health and disease in the global gut microbiome. Adv Sci (Weinh). (2026) 13(4):e17087. doi: 10.1002/advs.202517087 42153961 PMC13335992

