# Supplementary material for: Peripheral neurotransmitter metabolic signatures in schizophrenia: a targeted metabolomics study
Source: Front Psychiatry. 2026 Sep 16;17:1935417. doi: 10.3389/fpsyt.2026.1935417 (PMC13624068; doi:10.3389/fpsyt.2026.1935417)
Supplement: Supplementary file 1 [file Table1.docx]

Supplementary Table S1. Major Experimental Instruments

| Instrument | Model | Manufacturer |
| --- | --- | --- |
| Ultra‑performance liquid chromatography system | 1290 Infinity UPLC | Agilent Technologies |
| Triple quadrupole mass spectrometer | 6500+ QTRAP | SCIEX |
| Centrifuge | 5430R | Eppendorf |
| Tissue homogenizer | JXFSTPRT‑CL | Shanghai Jingxin Industrial Development Co., Ltd. |
| Electronic balance | AL104 | METTLER TOLEDO |
| Vortex mixer | QT‑1 | Shanghai Qite Analytical Instrument Co., Ltd. |
| Ultrasonic cleaner | JP‑100 | Shenzhen Jiemeng Cleaning Equipment Co., Ltd. |
| Chromatography column | ACQUITY UPLC BEH Amide, 1.7 μm, 2.1 mm × 150 mm | Waters |
